# Microstructures, Hardness and Corrosion Behaviors of FeCoNiNb_0.5_Mo_0.5_ and FeCoNiNb High-Entropy Alloys

**DOI:** 10.3390/ma11010016

**Published:** 2017-12-23

**Authors:** Chun-Huei Tsau, Wei-Li Wang

**Affiliations:** Institute of Nanomaterials, Chinese Culture University, Taipei 111, Taiwan; willy11424@yahoo.com.tw

**Keywords:** FeCoNiNb_0.5_Mo_0.5_, FeCoNiNb, microstructure, hardness, corrosion

## Abstract

This study investigates the effects of niobium and molybdenum on FeCoNi alloy, including on the microstructures and hardness of FeCoNiNb_0.5_Mo_0.5_ and FeCoNiNb alloys, and the polarization behaviors of these alloys in 1 M sulfuric acid and 1 M sodium chloride solutions. The results in this study indicate that both FeCoNiNb_0.5_Mo_0.5_ and FeCoNiNb alloys had a dual-phased dendritic microstructure; all of the phases in these alloys were solid solution phases, and no ordering was observed. Therefore, the solid solution effect significantly increased the hardness of these two alloys; in particular, FeCoNiNb alloy had the highest hardness of the alloys of interest. The corrosion resistance of FeCoNiNb_0.5_Mo_0.5_ and FeCoNiNb alloys was less than that of FeCoNi alloy because of their dual-phased dendritic microstructures. The corrosion resistance of the FeCoNiNb_0.5_Mo_0.5_ alloy exceeded that of the FeCoNiNb alloy in these solutions. However, FeCoNiNb_0.5_Mo_0.5_ and FeCoNiNb alloys exhibited a favorable combination of corrosion resistance and hardness.

## 1. Introduction

High-entropy alloys [1,2] with unique properties have recently become increasingly important. Corrosion resistance is also an important property of metals for structural application. The high-entropy alloy concept is used to develop corrosion-resistant alloys [3,4,5]. This study develops high-entropy alloys with a combination of good corrosion resistance and hardness. The FeCoNi alloy has a very good corrosion resistance in 1 M deaerated sulfuric acid and 1 M deaerated sodium chloride solutions [6]. However, the softness of FeCoNi alloy, HV 112, limits its structural applications. The goal of this work is to improve the hardness of FeCoNi alloy while maintaining its corrosion resistance. For example, the addition of a suitable amount of niobium can increase the corrosion resistance of Fe_73.5_Cu_1_Nb_x_Si_13.5_B_10_ alloys [7]; and adding molybdenum can increase the corrosion resistance of Ni-Cr-Mo-Fe alloys in H_2_S–Cl^−^ environments because Mo improves the localized corrosion resistance [8,9]. Therefore, in this study, Nb and Mo are used as the fourth and fifth elements to enhance the properties of FeCoNiNb_0.5_Mo_0.5_ and FeCoNiNb alloys. The microstructural evolutions of these alloys, and the effect on the polarization behaviors of adding Nb and Mo into these alloys in 1 M sulfuric acid and 1 M sodium chloride solutions are investigated.

## 2. Experimental

FeCoNiNb_0.5_Mo_0.5_ and FeCoNiNb alloys were prepared by arc melting using appropriate amounts of the constituent elements with purities above 99.9%. The alloys were made by combining the above elements and arc-melting them in an argon atmosphere with a partial pressure of 200 torr. Table 1 presents the chemical compositions of the alloys. The microstructural evolution of the alloys was observed using a field emission scanning electron microscope with an energy dispersive spectrometer (SEM/EDS, JEOL JSM-6335, JEOL Ltd., Tokyo, Japan), which was operated at 15 kV. Samples for SEM observation were metallographically prepared and chemically etched in Marble’s etching solution (HCl 50 mL + CuSO_4_ 10 g + H_2_O 100 mL). The structures were characterized by X-ray diffraction (XRD) using a Rigaku ME510-FM2 (Rigaku Ltd., Tokyo, Japan) with Cu-Kα (with a wavelength of 1.541 Å) radiation, operated at 30 kV. The microstructures and lattice images of the alloys were obtained using a high-resolution transmission electron microscope (HREM, JEOL JEM-3000F, JEOL Ltd., Tokyo, Japan), which was operated at 300 kV. The corresponding selection area diffraction patterns (SAD) were obtained from the high-resolution lattice images by fast Fourier transformation (FFT) in Gatan digital micrograph software. Thin foil specimens for TEM observation were electrochemically prepared in a digital Fischione twin-jet electropolisher, model 110 (Fischione Instruments Co., Pittsburgh, PA, USA), in a solution of 10 vol.% perchloric acid and 90 vol.% methanol at a potential of 30 V. The hardness of the alloys was measured using both a Mitutoyo Akashi MVK-G1500 microhardness tester (Mitutoyo Co., Kanagawa, Japan) under a load of 10 gf and a Matsuzawa Seiki MV1 Vicker’s hardness tester (Matsuzawa Co., Akita, Japan) under a load of 30 kgf.

Polarization curves of the alloys were obtained in a potentiostat/galvanostat (Autolab PGSTAT302N, Metrohm Autolab B.V., Utrecht, The Netherlands) using a three-electrode system at a scanning rate of 1 mV/s. The polarization data were compared with those of commercial 304 stainless steel (304SS) whose composition was by weight 71.61% Fe, 18.11% Cr, 8.24% Ni, 1.12% Mn, 0.75% Si, 0.05% Co, 0.02% Mo, 0.05% C, 0.03% P, and 0.02% S. The as-cast FeCoNiNb_0.5_Mo_0.5_ and FeCoNiNb alloys for polarization testing were mounted in epoxy resin, and the exposed surface area of each was fixed at 0.1964 cm^2^ (with a diameter of 0.5 cm). The reference electrode was a saturated silver chloride electrode (Ag/AgCl), and the counter electrode was a smooth Pt sheet. All the potentials that are below a saturated silver chloride electrode (SSE), whose reduction potential is 222 mV higher than that of the standard hydrogen electrode (SHE) at 25 °C [10]. The specimens whose polarization curves were obtained were all mechanically wet-polished using 1200 SiC grit paper. Test solutions with a concentration of 1 M were prepared from reagent-grade sulfuric acid (H_2_SO_4_) and sodium chloride (NaCl) that were dissolved in distilled water. To eliminate any effect of dissolved oxygen, the solutions were deaerated by bubbling nitrogen gas through them before and during the polarization experiments.

## 3. Results and Discussion

**Figure 1:**
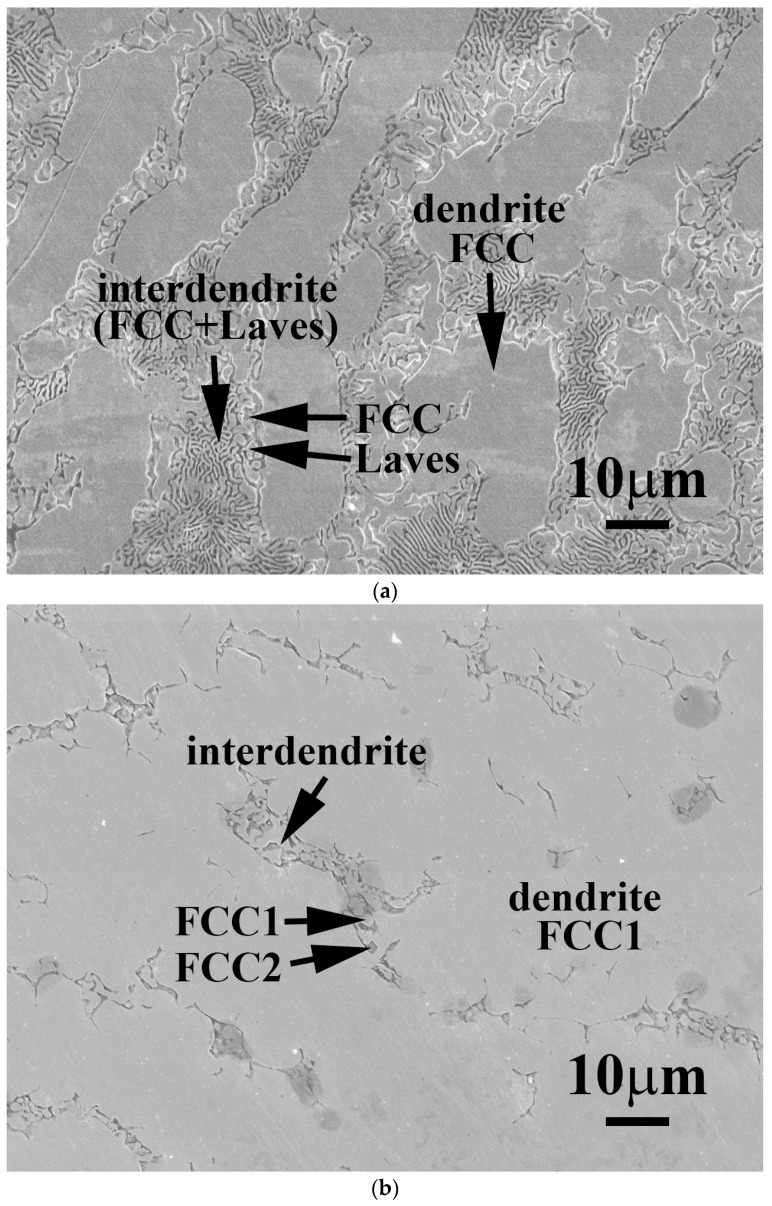
The microstructures of (**a**) FeCoNiNb_0.5_Mo_0.5_ alloy; and (**b**) FeCoNiNb alloy.

**Figure 2:**
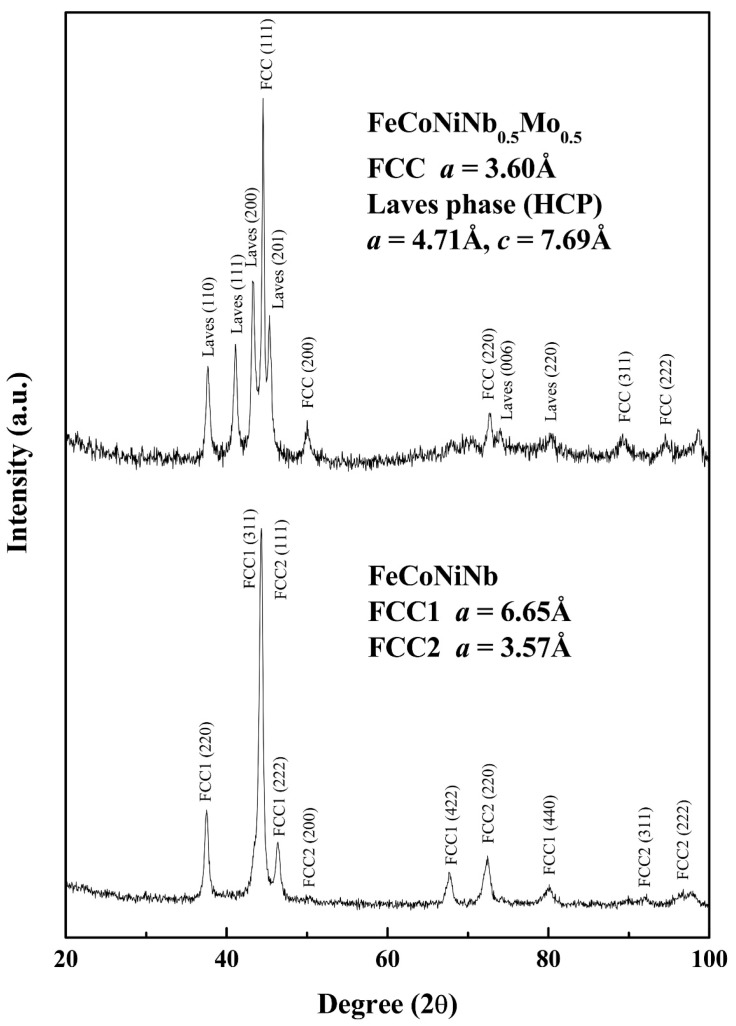
XRD patterns of FeCoNiNb_0.5_Mo_0.5_ alloy and FeCoNiNb alloy.

Figure 1 shows as-cast micrographs of FeCoNiNb_0.5_Mo_0.5_ and FeCoNiNb alloys, which reveal dendritic microstructures. The dendrites exhibit a single phase and the interdendritic regions exhibit a dual-phased eutectic structure. Hence, adding Nb or/and Mo would have changed their microstructures to dendritic, because FeCoNi alloy had a granular structure [6]. Figure 2 displays the XRD patterns of the FeCoNiNb_0.5_Mo_0.5_ and FeCoNiNb alloys. The two phases of the FeCoNiNb_0.5_Mo_0.5_ alloy were the laves phase (HCP) and the FCC phase. The lattice constants of the laves phase were *a_0_* = 4.71 and *c_0_* = 7.69 Å; the lattice constant of the FCC phase was 3.60 Å. The two phases of FeCoNiNb alloy are FCC1 and FCC2. The lattice constants of the FCC1 and FCC2 phases herein were 6.65 and 3.57 Å, respectively. The structures of the phases of FeCoNiNb_0.5_Mo_0.5_ and FeCoNiNb alloys were identified by comparing their SEM micrographs and the intensities of their XRD peaks. The dendrites in each alloy had higher XRD intensities because of their volume fractions. Therefore, the dendrites of FeCoNiNb_0.5_Mo_0.5_ alloy were an FCC phase, and the dendrites of the FeCoNiNb alloy were an FCC1 phase.

**Figure 3:**
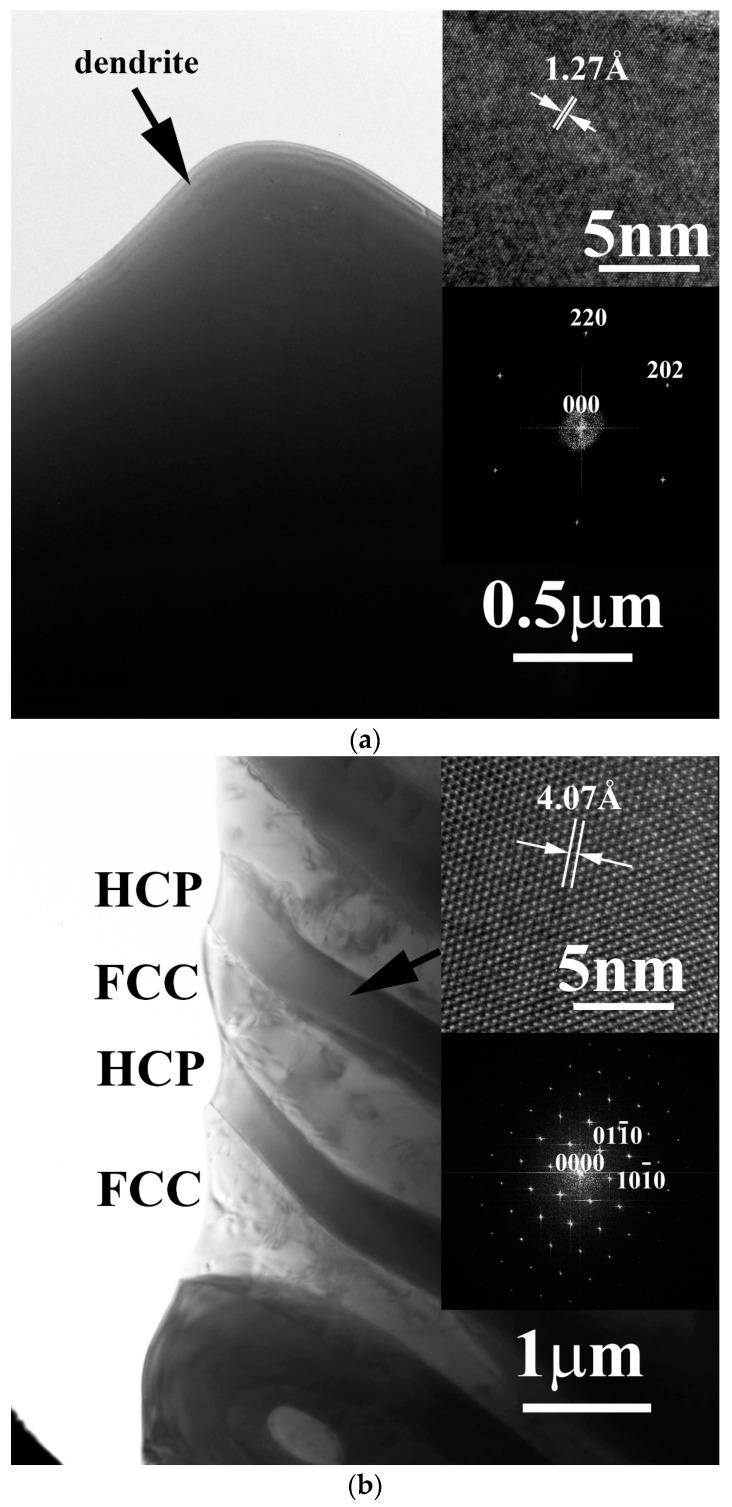

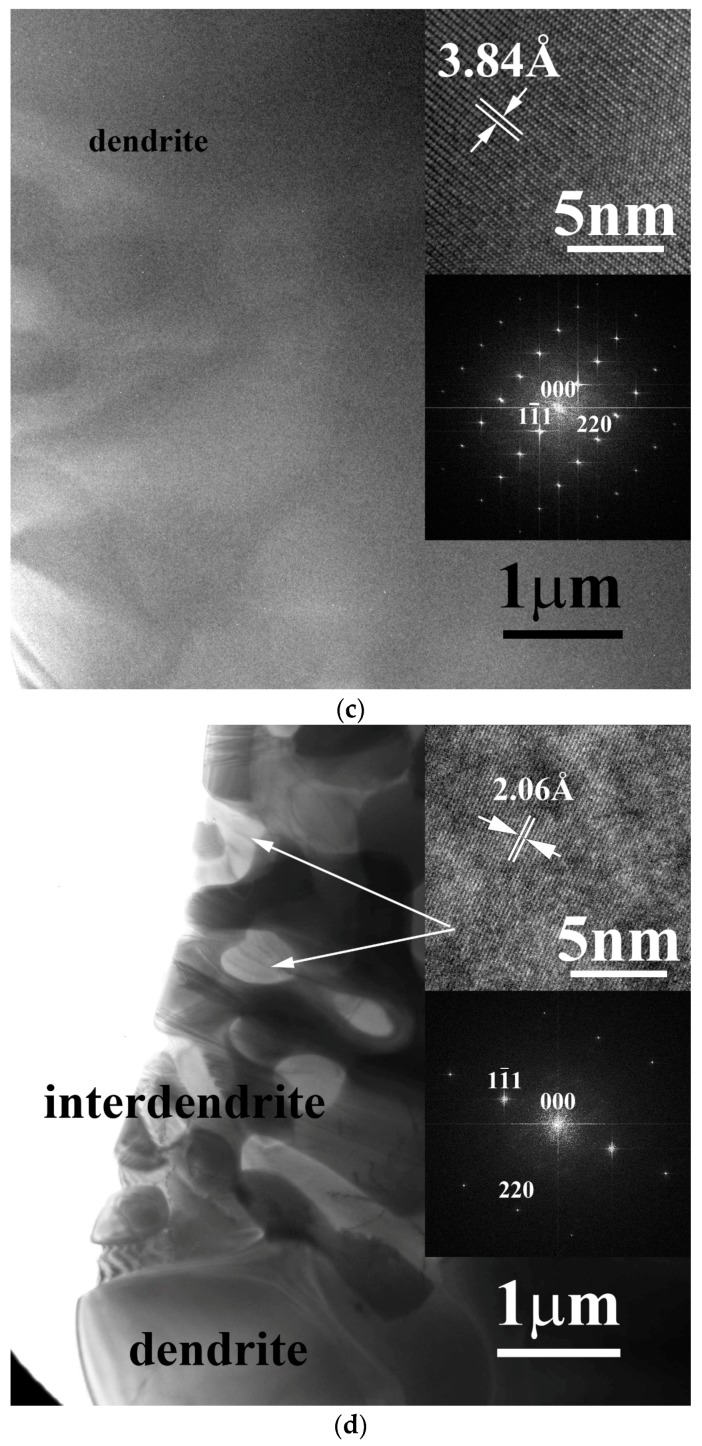
TEM images of the phases in FeCoNiNb_0.5_Mo_0.5_ and FeCoNiNb alloys, and the corresponding lattice images and FFT diffraction patterns, (**a**) the dendrite of FeCoNiNb_0.5_Mo_0.5_ alloy; (**b**) the laves phase in the interdendrite of FeCoNiNb_0.5_Mo_0.5_ alloy; (**c**) the dendrite (FCC1 phase) of FeCoNiNb alloy; and (**d**) the FCC2 phase in the interdendrite of FeCoNiNb alloy.

The phases of the FeCoNiNb_0.5_Mo_0.5_ alloy and FeCoNiNb alloys were all identified by TEM, as shown in Figure 3. The micrograph of each phase in the FeCoNiNb_0.5_Mo_0.5_ alloy and FeCoNiNb alloys was examined carefully, and the FFT diffraction pattern of each phase was obtained from its high-resolution lattice images. Figure 3a shows a TEM image of an FCC dendrite of FeCoNiNb_0.5_Mo_0.5_ alloy, and the insets present the corresponding lattice image and FFT diffraction pattern, which indicates that the image was taken with a beam direction of [111]. Figure 3b shows a TEM image of a HCP-structured laves phase in the interdendrite of FeCoNiNb_0.5_Mo_0.5_ alloy, which was obtained with a beam direction of [0001]. Figure 3c shows a TEM image of an FCC1 dendrite of FeCoNiNb alloy, which was obtained with a beam direction of [$\bar{1}$12]. Figure 3d shows a TEM image of an FCC2 phase in the interdendrite of FeCoNiNb alloy, which was obtained with beam direction of [$\bar{1}$12].

Table 2 presents the chemical compositions of the phases in the alloys. The Nb and Mo contents of the FCC-dendrite of FeCoNiNb_0.5_Mo_0.5_ alloy exceed those in the laves phase in the interdendritic region. The Nb content of the FCC1 dendrite of FeCoNiNb alloy exceeds that of the FCC2 phase in the interdendrite. The melting points of Fe, Co, Ni, Nb and Mo are 1536, 1498, 1453, 2415 and 2610 °C, respectively [10]. The elements Nb and Mo have higher melting points than the others. Therefore, a melt that contained more Nb and Mo solidified first to form the dendrites.

Table 3 presents the hardness of the FeCoNiNb_0.5_Mo_0.5_, FeCoNiNb, FeCoNi and 304SS alloys. FeCoNi alloy is very soft and has an HV of only 112, making it even softer than commercial 304 stainless steel, which has a hardness of HV 185. However, adding Nb/Mo to FeCoNi alloy significantly increased its hardness. FeCoNiNb_0.5_Mo_0.5_ and FeCoNiNb alloys had higher hardness values of HV 629 and HV 798, respectively. The microhardness values of the FCC-dendrite and eutectic-interdendrite of the FeCoNiNb_0.5_Mo_0.5_ alloy were HV 677 and HV 317, respectively. The microhardness values of the FCC1-dendrite and eutectic-interdendrite of FeCoNiNb alloy were HV 840 and HV 233, respectively, to which the solid solution effect contributed. The atomic radii of Fe, Co, Ni, Nb and Mo are 0.124, 0.125, 0.125, 0.143 and 0.140 nm, respectively [11]. The radii of Nb and Mo (exceeds OR are larger than) those of the other elements. No superlattice spot was observed in the TEM diffraction patterns indicating that all of the phases in FeCoNiNb_0.5_Mo_0.5_ and FeCoNiNb alloys were solid solution phases; a large lattice distortion was thus expected due to the presence of elements with larger radii, Nb and Mo. Therefore, the solid solution effect was the cause of the increased hardness.

**Figure 4:**
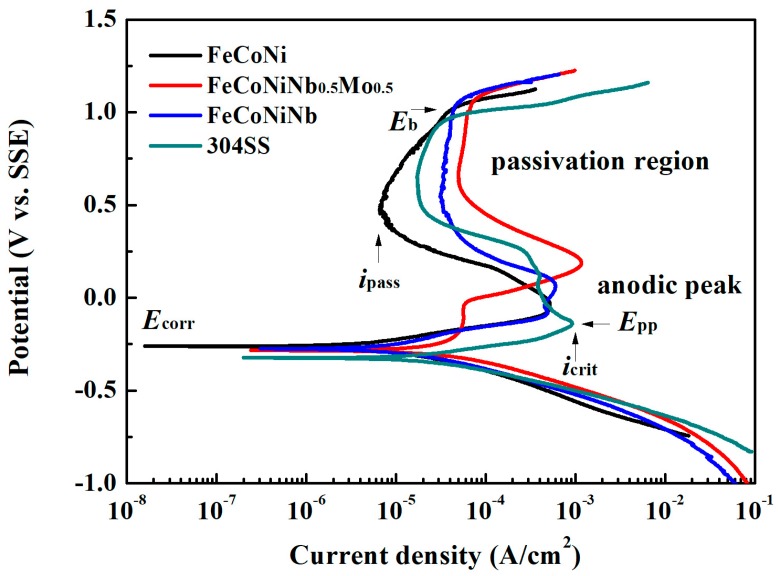
Polarization curves of FeCoNiNb_0.5_Mo_0.5_, FeCoNiNb, FeCoNi and 304SS alloys in 1 M deaerated H_2_SO_4_ solution at 30 °C.

Figure 4 plots the polarization curves of FeCoNiNb_0.5_Mo_0.5_, FeCoNiNb, FeCoNi and 304SS alloys in 1 M deaerated H_2_SO_4_ solution and indicates important characteristics, such as corrosion potential (*E*_corr_), passivation potential (*E*_pp_, the potential of the anodic peak), critical current density of the anodic peak (*i*_crit_), passive current density (*i*_pass_) and breakdown potential (*E*_b_). Table 4 presents the data that are obtained from the polarization curves. The corrosion current density (*i*_corr_) associated with each polarization curve was obtained from the intersection of the free corrosion potential and the cathodic Tafel line. The corrosion current densities of FeCoNiNb_0.5_Mo_0.5_ and FeCoNiNb alloys exceeded the *i*_corr_ of the single FCC-phased FeCoNi alloy, because they both had a dual-phased microstructure and thus easily formed local cells between the anodic and cathodic regions. However, the corrosion current densities of FeCoNiNb_0.5_Mo_0.5_ and FeCoNiNb alloys were smaller than the *i*_corr_ of 304 stainless steel. Therefore, the corrosion rates of FeCoNiNb_0.5_Mo_0.5_ and FeCoNiNb alloys were smaller than that of 304 stainless steel in 1 M deaerated H_2_SO_4_ solution at 30 °C. The corrosion potentials of FeCoNiNb_0.5_Mo_0.5_ and FeCoNiNb alloys were only slightly lower than the *E*_corr_ of FeCoNi alloy, but these alloys were still nobler than 304 stainless steel. However, the passive current densities of FeCoNiNb_0.5_Mo_0.5_ and FeCoNiNb alloys exceed those of 304 stainless steel and FeCoNi alloy.

**Figure 5:**
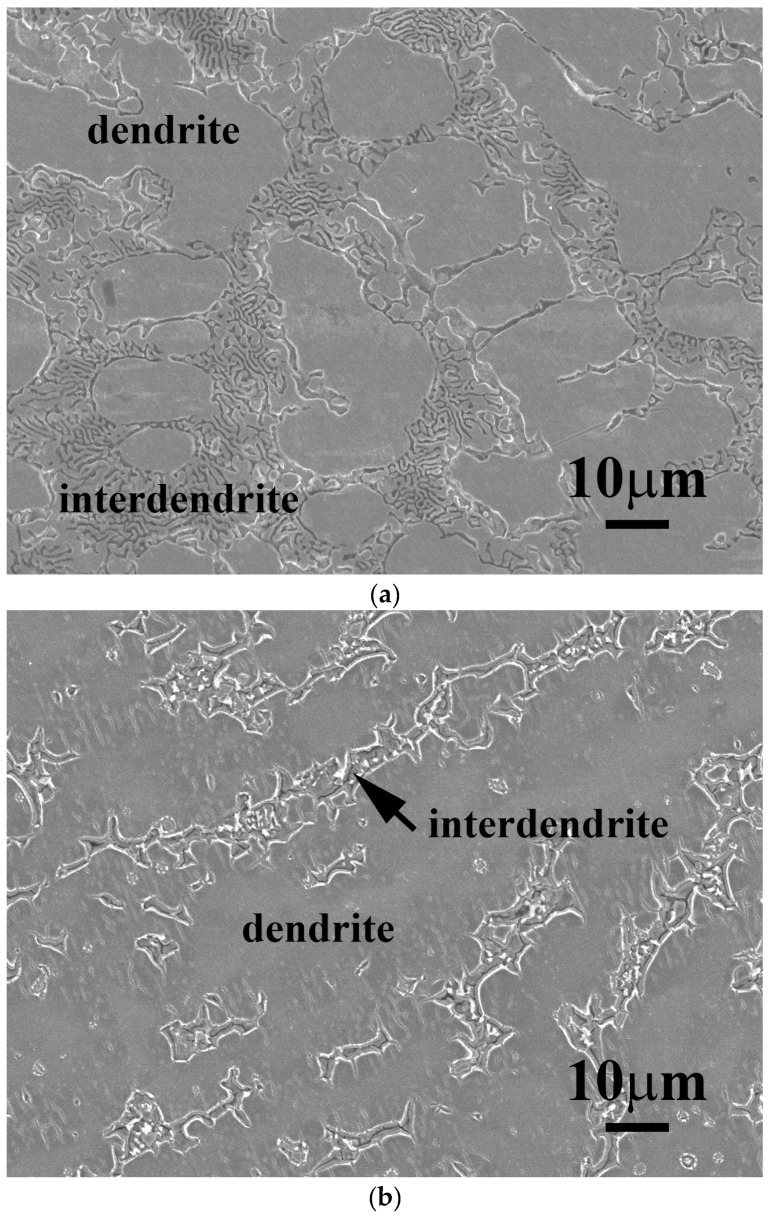
SEM micrographs of the alloys after polarization test in 1 M deaerated H_2_SO_4_ solution at 30 °C, (**a**) FeCoNiNb_0.5_Mo_0.5_ alloy; and (**b**) FeCoNiNb alloy.

Figure 5 shows the SEM micrographs of FeCoNiNb_0.5_Mo_0.5_ and FeCoNiNb alloys after the polarization test in 1 M deaerated H_2_SO_4_ solution at 30 °C. The dendrites of FeCoNiNb_0.5_Mo_0.5_ alloy were an FCC phase and the interdendrites were a eutectic structure of FCC and laves phases. The laves phase was the matrix of the interdendrite, which was corroded during the polarization test in 1 M deaerated H_2_SO_4_ solution at 30 °C, as shown in Figure 5a. In contrast, the morphology of the FCC phase of FeCoNiNb_0.5_Mo_0.5_ alloy was almost unchanged after the polarization test. Figure 5b shows a micrograph of FeCoNiNb alloy after a polarization test in 1 M deaerated H_2_SO_4_ solution at 30 °C. The dendrites of FeCoNiNb alloy were an FCC1 phase, and the interdendrites were a eutectic of FCC1 and FCC2 phases. The FCC2 phase in the interdendrite of FeCoNiNb alloy was the main corroded phase in the polarization test in 1 M deaerated H_2_SO_4_ solution at 30 °C. The FCC1 phase of FeCoNiNb alloy was only slightly corroded after the polarization test. Therefore, the main corroded areas of these two alloys are the interdendrites. Since the interdendrite area of FeCoNiNb alloy was less than that of FeCoNiNb_0.5_Mo_0.5_ alloy, the corrosion current density of FeCoNiNb alloy was less than that of FeCoNiNb_0.5_Mo_0.5_ alloy. 

**Figure 6:**
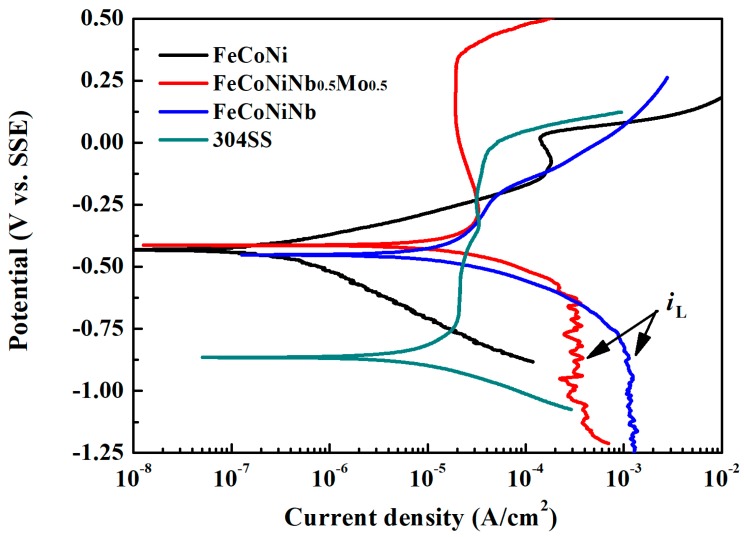
Polarization curves of FeCoNi, FeCoNiNb_0.5_Mo_0.5_, FeCoNiNb and 304SS alloys in 1 M deaerated NaCl solution at 30 °C.

Figure 6 plots the polarization curves of FeCoNiNb_0.5_Mo_0.5_, FeCoNiNb, FeCoNi and 304SS alloys in 1 M deaerated NaCl solution. Table 5 presents the corrosion potentials and corrosion current densities that are obtained from the curves. The corrosion potentials of FeCoNiNb_0.5_Mo_0.5_ and FeCoNiNb alloys were close to the *E*_corr_ of FeCoNi alloy, these alloys were all nobler than 304 stainless steel. This result indicates that adding Nb/Mo to FeCoNi alloy did not significantly change its *E*_corr_, although but did change its microstructures to dual-phased ones. However, the dual-phased microstructures of FeCoNiNb_0.5_Mo_0.5_ and FeCoNiNb alloys significantly influenced on the *i*_corr_ of the alloys. The corrosion current densities of FeCoNiNb_0.5_Mo_0.5_ and FeCoNiNb alloys exceeded the *i*_corr_ of FeCoNi alloy; but smaller than the *i*_corr_ of 304 stainless steel. FeCoNiNb_0.5_Mo_0.5_ alloy had a large passivation region because of the addition of molybdenum. Adding Mo can reportedly increase the corrosion resistance of the alloy in a solution that contains chloride ions [9] because molybdenum can increase the stability of the passivation films of steels. In contrast, the corrosion resistance of FeCoNiNb alloy in 1 M NaCl solution was less than that of FeCoNiNb_0.5_Mo_0.5_ alloy. The cathodic limiting current densities (*i*_L_) in FeCoNiNb_0.5_Mo_0.5_ and FeCoNiNb alloys were observed. The cathodic limiting current density corresponds to the maximum reaction rate, which is herein limited by the diffusion rate of hydroxyl ions (OH^−^) in solution [10]. However, no cathodic limiting current density was observed in FeCoNi alloy [6].

**Figure 7:**
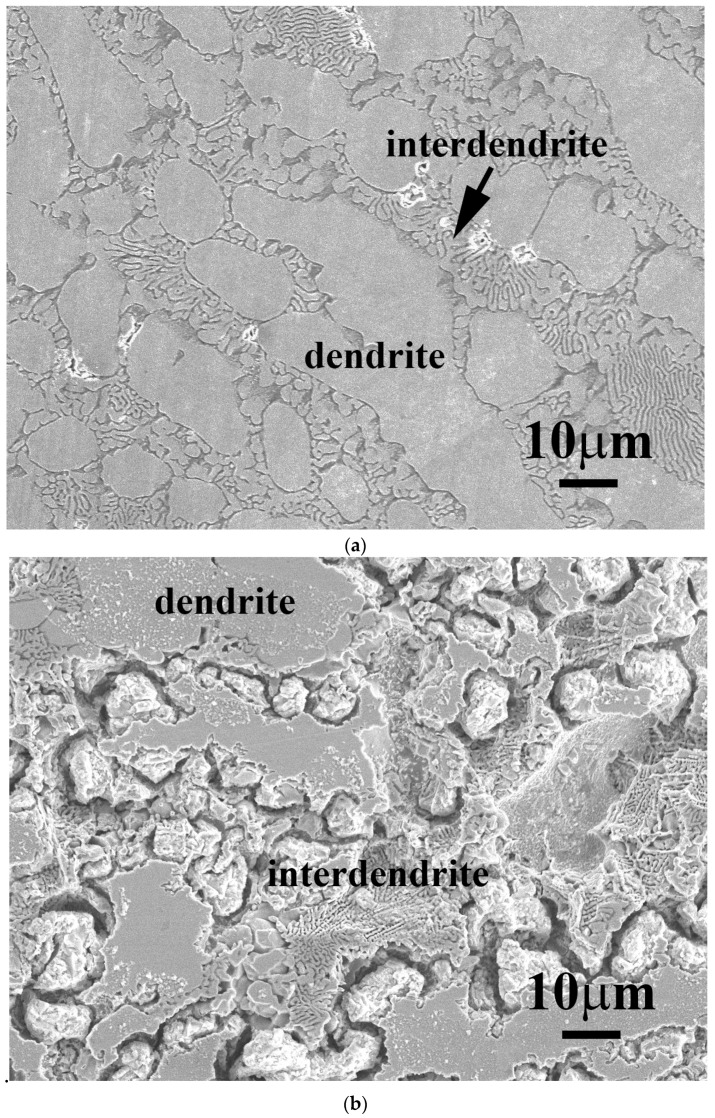
SEM micrographs of the alloys after polarization test in 1 M deaerated NaCl solution at 30 °C, (**a**) FeCoNiNb_0.5_Mo_0.5_ alloy; and (**b**) FeCoNiNb alloy.

Figure 7 shows SEM micrographs of FeCoNiNb_0.5_Mo_0.5_ and FeCoNiNb alloys after the polarization test in 1 M deaerated NaCl solution at 30 °C. As for the FeCoNiNb_0.5_Mo_0.5_ and FeCoNiNb alloys that were tested in 1 M deaerated H_2_SO_4_ solution, their main corroded areas were the interdendrites. Figure 7a shows a micrograph of FeCoNiNb_0.5_Mo_0.5_ alloy after the polarization test. The corroded phase was the laves phase in the interdendrite of FeCoNiNb_0.5_Mo_0.5_ alloy. The FCC-structured dendrite and FCC phase in the interdendrite were not corroded and so their original morphologies were retained. Figure 7b shows a micrograph of FeCoNiNb alloy after the polarization test. The FCC2 phase in the interdendrite of FeCoNiNb alloy was the main corroded area. The FCC1 dendrite of FeCoNiNb alloy was also corroded, but to a lesser extent than the FCC2 phase.

## 4. Conclusions

1. Both FeCoNiNb_0.5_Mo_0.5_ and FeCoNiNb alloys had dual-phased dendritic structures. The phases in FeCoNiNb_0.5_Mo_0.5_ alloy were FCC and laves phases, and the dendrites exhibited an FCC phase. The phases in FeCoNiNb alloy were FCC1 and FCC2 phases, and the dendrites exhibited an FCC1 phase.
2. The hardness of FeCoNiNb alloy was higher than that FeCoNiNb_0.5_Mo_0.5_ alloy; and both FeCoNiNb_0.5_Mo_0.5_ and FeCoNiNb alloys were harder than FeCoNi alloy and 304 stainless steel.
3. The corrosion resistance of FeCoNiNb_0.5_Mo_0.5_ alloy exceeded that of FeCoNiNb alloy in 1 M H_2_SO_4_ and 1 M NaCl solutions. The corrosion resistance of these two alloys was less than that of FeCoNi alloy but greater than that of 304 stainless steel in these two solutions.

## Figures and Tables

**Table 1:** Chemical compositions of FeCoNiNb_0.5_Mo_0.5_ and FeCoNiNb alloys analyzed by SEM/EDS.

Alloys	Compositions (at.%)				
	Fe	Co	Ni	Nb	Mo
FeCoNiNb_0.5_Mo_0.5_	24.6 ± 1.3	22.9 ± 1.4	22.6 ± 0.5	15.1 ± 0.3	14.8 ± 0.9
FeCoNiNb	23.8 ± 0.3	24.3 ± 0.5	23.1 ± 0.7	28.8 ± 0.6	N/A

**Table 2:** Phase constitutions of the FeCoNiNb_0.5_Mo_0.5_ and FeCoNiNb alloys analyzed by SEM/EDS.

Alloys and Phases	Compositions (at.%)				
	Fe	Co	Ni	Nb	Mo
FeCoNiNb_0.5_Mo_0.5_					
FCC (dendrite)	22.5 ± 0.3	23.9 ± 1.0	17.6 ± 0.5	18.5 ± 0.6	17.5 ± 1.0
laves (in interdendrite)	26.0 ± 1.3	23.3 ± 0.8	30.5 ± 2.5	9.5 ± 1.0	10.8 ± 1.3
FeCoNiNb					
FCC1 (dendrite)	23.7 ± 0.9	24.1 ± 0.8	20.8 ± 1.0	31.5 ± 0.7	N/A
FCC2 (in interdendrite)	30.0 ± 1.4	22.4 ± 0.5	31.2 ± 1.5	16.7 ± 0.9	N/A

**Table 3:** Hardness (HV) of FeCoNiNb_0.5_Mo_0.5_, FeCoNiNb, FeCoNi and 304SS alloys.

Alloy	Overall Load: 30 kgf	Dendrite Load: 10 gf	Interdendrite Load: 10 gf
FeCoNiNb_0.5_Mo_0.5_	629 ± 7	677 ± 12	317 ± 7
FeCoNiNb	798 ± 8	840 ± 24	233 ± 12
FeCoNi	112 ± 2	N/A	N/A
304SS	185 ± 2	N/A	N/A

**Table 4:** Polarization data (*i*_corr_ and *E*_corr_) of FeCoNiNb_0.5_Mo_0.5_, FeCoNiNb, FeCoNi and 304SS alloys in 1 M H_2_SO_4_ solution at 30 °C.

Alloys	*i*_corr_ μA/cm^2^	*E*_corr_ V vs. SSE	*E*_pp_ V vs. SSE	*i*_crit_ mA/cm^2^	*i*_pass_ μA/cm^2^
FeCoNiNb_0.5_Mo_0.5_	41.0	−0.283	0.189	1.170	49.7
FeCoNiNb	15.7	−0.274	0.058	0.600	31.7
FeCoNi	10.6	−0.261	−0.035	0.650	6.6
304SS	30.0	−0.320	−0.140	0.930	17.2

**Table 5:** Polarization data of FeCoNi, FeCoNiNb_0.5_Mo_0.5_, FeCoNiNb and 304SS alloys in 1 M NaCl solution at 30 °C.

Alloys	*i*_corr_	*E*_corr_
	μA/cm^2^	V vs. SSE
FeCoNiNb_0.5_Mo_0.5_	33.1	−0.413
FeCoNiNb	43.0	−0.452
FeCoNi	0.4	−0.451
304SS	70.0	−0.860

## References

[1] Yeh J.W., Chen S.K., Lin S.J., Gan J.Y., Chin T.S., Shun T.T., Tsau C.H., Chang S.Y. (2004). Nanostructured high-entropy alloys with multiple principal elements: Novel alloy design concepts and outcomes. Adv. Eng. Mater..

[2] Murty B.S., Yeh J.W., Ranganathan S. (2014). High-Entropy Alloys.

[3] Qiu X.W., Zhang Y.P., He L., Liu C.G. (2013). Microstructure and corrosion resistance of AlCrFeCuCo high entropy alloy. J. Alloy Compd..

[4] Ren B., Liu Z.X., Li D.M., Shi L., Cai B., Wang M.X. (2011). Corrosion behavior of CuCrFeNiMn high entropy alloy system in 1 M sulfuric acid solution. Mater. Corros..

[5] Qiu Y., Gibson M.A., Fraser H.L., Birbilis N. (2015). Corrosion characteristics of high entropy alloys. Mater. Sci. Technol..

[6] Tsau C.H., Lin S.X., Fang C.H. (2017). Microstructures and corrosion behaviors of FeCoNi and CrFeCoNi equimolar alloys. Mater. Chem. Phys..

[7] Mariano N.A., Souza C.A.C., May J.E., Kuri S.E. (2003). Influence of Nb content on the corrosion resistance and saturation magnetic density of FeCuNbSiB alloys. Mater. Sci. Eng. A.

[8] Tomio A., Sagara M., Doi T., Amaya H., Otsuka N., Kudo T. (2015). Role of alloyed molybdenum on corrosion resistance of austenitic Ni–Cr–Mo–Fe alloys in H_2_S–Cl^−^ environments. Corros. Sci..

[9] Hashimoto K., Asami K., Teramoto K. (1979). An X-ray photo-electron spectroscopic study on the role of molybdenum in increasing the corrosion resistance of ferritic stainless steels in HCl. Corros. Sci..

[10] Revie R.W., Uhlig H.H. (2008). Corrosion and Corrosion Control.

[11] Smith W.F. (2004). Foundations of Materials Science and Engineering.

